# Influence of Atrioventricular Nodal Reentrant Tachycardia Ablation on Right to Left Inter-atrial Conduction

**Published:** 2005-10-01

**Authors:** Abdurrahman Eksik, Ahmet Akyol, Tugrul Norgaz, Izzet Erdinler

**Affiliations:** Siyami Ersek Thoracic and Cardiovascular Surgery Center, Cardiology Department, Istanbul-Turkey

**Keywords:** RF ablation, AVNRT, inter-atrial conduction time

## Abstract

**Background:**

Radiofrequency (RF) catheter ablation is the procedure of choice for the potential cure of atrioventricular nodal reentrant tachycardia (AVNRT) with high success rates. We hypothesed that as a result of the close proximity of Koch’s triangle and low inter-atrial septal fibers, the RF ablation applied at this region may result in prolongation of inter-atrial conduction time (IACT).

**Methods:**

RF ablation of AVNRT was performed by conventional technique. IACT was measured before and 20 minutes after RF ablation during sinus rhythm. Number of ablations given and duration of ablation were noted.

**Results:**

The study group was consisted of 48 patients (36 [75%] female, 12 [25%] male, mean age 43.4 ± 14. 5 years). RF ablation was successful in all patients. Mean RF time was 4. 0 ± 3. 3 minutes and mean number of RF was 11. 9 ± 9, 8. The mean IACT was 70.1 ± 9.0 ms before ablation and 84.9 ± 12.7 ms after ablation, which demonstrated a significant prolongation (p<0.001). The prolongation of IACT was very well correlated with the number of (r=0.897, p<0.001) and duration of RF (r=0.779; p<0.001).

**Conclusions:**

RF ablation of AVNRT results in prolongation of IACT. The degree of prolongation is associated with the duration and number of RF ablations given. The relationship between this conduction delay and late arrhythmogenesis need to be evaluated.

## Introduction

Radiofrequency (RF) catheter ablation is the procedure of choice for the potential cure of atrioventricular nodal reentrant tachycardia (AVNRT) with high success rates. Slow pathway (SP) ablation is the best method for the treatment of AVNRT. It has been shown that successful atrial flutter ablation was associated with an altered sequence of left atrial activation [1]. But, no studies have evaluated the effect of RF ablation of AVNRT on inter-atrial conduction. We hypothesed that as a result of the close proximity of Koch’s triangle and low inter-atrial septal fibers, the RF ablation applied at this region may result in prolongation of inter-atrial conduction time (IACT). For this purpose, we evaluated the effect of RF ablation on IACT before and after ablation of SP, during sinus rhythm.

## Materials and Methods

### Patients

The study group was consisted of 48 consecutive patients with common type AVNRT who had inducible AVNRT with dual or multiple atrioventricular node (AVN) physiology in the atrial extra-stimulations and who had undergone SP ablation between April 2004 and March 2005. Exclusion criteria were presence of clinically significant valvular, congenital or ischemic heart disease, any type of cardiomyopathy, and heart failure and patients with dilatated atria. After informed consent was obtained, the electrophysiologic study (EPS) was performed in the fasting state without any sedative medications. Antiarrhythmic drugs were discontinued for at least 5 half-lives before the ablation. Transthoracic echocardiography was performed in all patients to determine the presence of exclusion criteria.

### Catheters and Electrogram Recordings

Three 5F quadripolar electrode catheters were introduced via the femoral vein, and were positioned against the high right atrium (HRA), the His bundle region, and the right ventricular apex (RVA) under fluoroscopic guidance. One 7F decapolar catheter (inter-electrode distance: 2mm) was introduced into the coronary sinus via the femoral vein. Patients’ data were analyzed on a paper recording at 200 mm/s (Model VR-13, Biomedical Systems, USA), and data were stored for analysis on optical disks using a computer-recording system and were analyzed on screen (Cardio Lab System, Marquette, MI,USA).

### Basic Study and Diagnosis

Single extra-stimulation and incremental pacing were performed at the HRA and RVA sites for the basic electrophysiological evaluation. Prolongation of the atria-His (AH) interval of not less than 50 ms in response to the shortening of the coupling interval of the premature atrial stimulus by 10 ms was defined as the 'jump-up' phenomenon of the AH interval [2]. When the single atrial stimulus could not induce the AH jump-up, a double-atrial-extra-stimulus protocol was performed to achieve shorter premature coupling intervals [3]. AVNRT was diagnosed in accordance with the following criteria: (1) the jump-up phenomenon of the AH interval after single or double atrial stimulus, (2) induction of narrow QRS regular tachycardia without the participation of an accessory pathway, and (3) simultaneous activation of the atrium and ventricle during tachycardia [4]. The second criterion was confirmed by single-ventricle scanning (ventricular pacing during His refractoriness in order to observe retrograde atrial activation) during tachycardia and sinus rhythm to exclude orthodromic AV reciprocal tachycardia involving an accessory pathway. When tachycardia could not be induced in the basic state, atropine was infused intravenously at a dose 1mg to increase the basic sinus rate by 20-50%, and the same stimulation protocol was repeated.

### Inter-atrial Conduction Time Measurement

The 5F quadripolar electrode catheter was positioned at HRA during sinus rhythm where the earliest RA activation was achieved. Right to left IACT’s were measured from earliest right atrial activation to the distal coronary sinus. Left to right IACT’s were measured from distal coronary sinus pacing to earliest right atrial activation. Measurements were done before ablation and 20 minutes after ablation. In order to be sure for the exact position of catheter during measurements before and after ablation, we stored the image of catheter position before ablation and used this image to guide the position of the catheter for measurement after ablation. In order to exclude the autonomic effects produced by RF energy after the procedure IACT measurement was repeated following atropine.

### SP Ablation

After the basic EPS, a 7F quadripolar steerable ablation catheter with 4 mm-tip and 2.5 mm interelectrode spacing (Marinr, Medtronic, USA) was introduced through the femoral vein. The catheter tip was initially positioned along the tricuspid annulus anterior to the ostium of the CS. In the lowest one-third of the area between the recording site at the His bundle and the ostium of the coronary sinus, the optimal ablation site was determined under guidance of the SP potential as described by Jackman et al5 with an A/V ratio of 0.1 to 0.5. Radiofrequency energy was delivered with a temperature controlled ablation unit (Atakr RF Ablation system, Medtronic) at 60°C during sinus rhythm. If a junctional beat was recognized within 10s, the energy delivery was continued for 1 min, and was terminated immediately in the cases showing impedance rise or any signs of AH block. After each ablation procedure, the pacing protocol was repeated to evaluate the inducibility of AVNRT. When AVNRT was still inducible, the ablation catheter was repositioned to a more superior region along the tricuspid annulus and the ablation procedure was continued. The ablation procedure was considered to be successful when AVNRT could not be induced 20 min after the last delivery of radiofrequency energy. In the post-ablation study, atropine was infused only when it had been needed for AVNRT induction in the basic state before the ablation. Single atrial echo beat or jump-up of the AH interval was allowed to remain.

### Statistical analysis

All conduction times were given in milliseconds and expressed as mean ± Standard deviation. Statistical analyses of data were performed using Student’s t test for paired data. Spearman’s correlation analyses were used to evaluate correlation between parameters. All analyses were performed by using SPSS 10.0 computer programme.A p value of < 0.05 was considered as statistically significant.

## Results

The study group was consisted of 48 patients (36 [75 %] female, 12 [25 %] male, mean age 43. 4 ± 14 and 5 years). RF ablation was successful in all patients. We observed no major complications. Mean RF time was 4. 0 ± 3.3 minutes and mean number of RF was 11. 9 ± 9.8 including very short RF’s to confirm nodal beat formation. Tachycardia was initiated by atrial extra stimulus in 43 patients and by atropine infusion in 5 patients.

**Figure 1:**
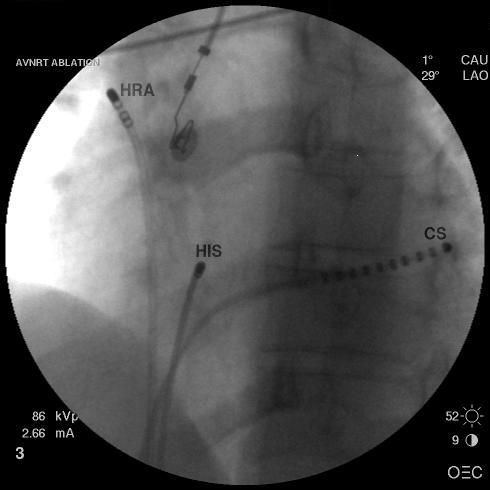
Left anterior oblique radiograph of catheters position during measurements

**Figure 2:**
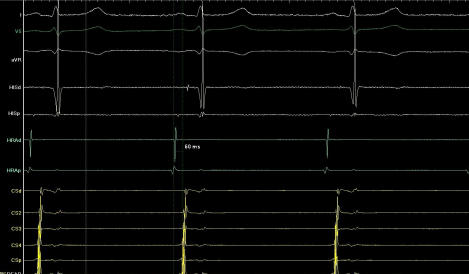
Right to left interatrial conduction time(IACT) before ablation. Earliest right atrial activation to the distal coronary sinus was measured as 60 msec.

**Figure 3:**
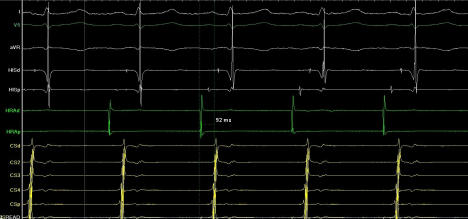
Right to left interatrial conduction time(IACT) after ablation. Earliest right atrial activation to the distal coronary sinus was measured as 92 msec.

**Figure 4:**
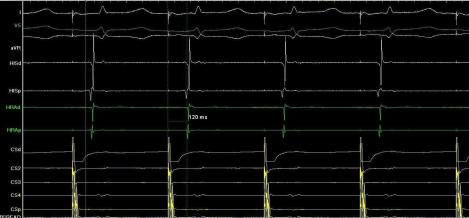
Left to right interatrial conduction time(IACT) before ablation. Left to right IACT’s were measured from distal coronary sinus pacing to earliest right atrial activation was measured as 120 msec.

**Figure 5:**
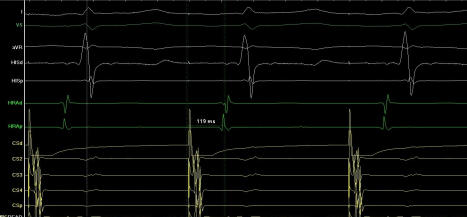
Left to right interatrial conduction time (IACT) after ablation. Left to right IACT’s were measured from distal coronary sinus pacing to earliest right atrial activation was measured as 119 msec.

Post ablation cycle length was 685.8 ± 81.0 ms. The sinus P wave morphology on 12-lead surface ECG was unchanged after ablation. The mean IACT was 70.1 ± 9.0 ms before ablation and 84.9 ± 12.7 ms after ablation, which demonstrated a significant prolongation (p<0.001). Sample intracardiac ECG tracings and fluoroscopic figures showing the position of catheters are presented in Figures 1-5.

**Figure 6:**
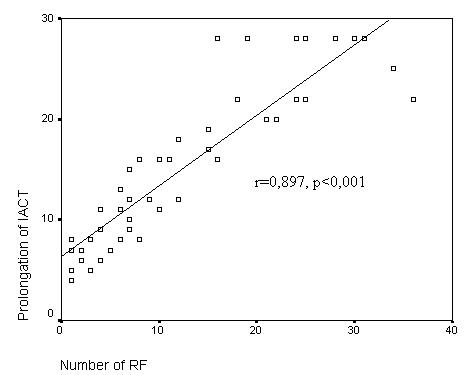
Association of IACT (Interatrial conduction time) prolongation (in miliseconds) and number of RF (radiofrequency) ablation

**Figure 7:**
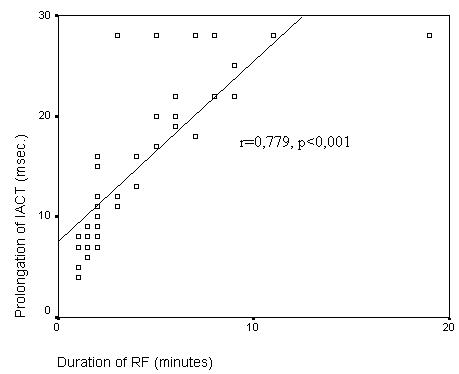
Association of IACT (Interatrial conduction time) prolongation (in miliseconds) and duration of RF (radiofrequency) ablation.

Left to right interatrial conduction time during pacing of distal coronary sinus was unchanged before and after ablation. The prolongation of IACT was very well correlated with the number of RF (r=0.897, p<0.001) and duration of RF (r=0,779; p<0. 0 01)(Figures 6-7). After ablation no IACT change was observed in repeated measurements in which atropine was given (the mean IACT was 84.9±12.7 ms after ablation and 83.4 ± 10.1 ms after atropine, p=0.884).

## Discussion

The atrioventricular (AV) junction is a complex anatomic structure located within an area called Koch’s triangle [6,7]. Koch’s triangle is bounded anteriorly and superiorly by the tendon of Todaro, posteriorly by the coronary sinus, and inferiorly by the annulus fibrosus of the tricuspid ring. The base of the triangle is marked by the ostium of the coronary sinus. SP conduction is registered near the ostium of the coronary sinus.

In adults, the compact AV node is relatively uniform in size, with a length of 5 to 7 mm and a width of 2 to 5 mm [8]. A greater variability in the size of Koch’s triangle was observed in the intraoperative and postmortem studies [9,10]. Fluoroscopic measurement with coronary sinus angiography also found a marked variation in the triangle’s dimensions [11]. In the right oblique (RAO) view, the distance between the His potential recording site and the floor of coronary sinus ostium was 25.9 + 7.9 mm. Marked differences in the arrangement of the superficial atrial muscle fibers in the area of the triangle of Koch have been reported in normal hearts [12]. Systematic anatomic investigation of the AV node in patients with AVNRT is lacking [13].

In the RAO view, the posteromedial tricuspid annulus between the level of the coronary sinus ostium and the His potential recording site was divided anatomically into posterior, median, and anterior zones. Energy was delivered along the tricuspid annulus, starting at the most posterior site, the floor of the coronary sinus ostium, and progressing to the most anterior site, just inferior to the His potential recording site. The inducibility of the AVNRT was assessed after each likely successful application. If the tachycardia was still inducible after two radiofrequency energy applications within each of the anatomic zones, the process was repeated. With this approach, the SP was successfully ablated in 188 (97%) of 193 patients [14].

Activation of the left atrium (LA) is complicated. Three routes of intra-atrial conduction are possible: (a) superiorly through Bachman’s bundle, (b) through the mid-atrial septum at the fossa ovalis, and (c) at the region of the central fibrous trigone at the apex of triangle of Koch. Activation of the left atrium over Bachman’s bundle can be observed in 50% - 70% of patients [15]. During sinus rhythm, two wave fronts depolarize the left atrium: one antero-lateral emerging from the Bachman’s bundle and the anterior aspect of the fossa ovalis, and one posterior proceeding from the low inter atrial septum [16,17]. But, Bachman’s bundle can not be differentiated from the circular fibers of the anterior wall or it is not prominent in some subjects [18]. As a result, the impulse does not move properly across the anterior wall from right to left atrium. Besides, Bachman’s bundle is not the only pathway connecting right and left atriums [19,19]. Although Bachmann’s bundle appeared to be the predominant inter-atrial connection, technical limitations may have reduced the accuracy of mapping in the posteroseptal LA and the region of the right inferior pulmonary vein ostium [20].

Markides et al. have described characteristic preferential activation patterns in the human LA. They have showed that, posterior inter-atrial connections, as described in recent studies of human atrial anatomy, were found to be at least as important as Bachmann’s bundle in right-to-left interatrial conduction during SR [21]. As a result, Markides et al. have shown that there are indeed multiple connections capable of right-to-left atrial conduction and that posterior communications play a major role, in contrast to left-to-right conduction. They also reported that the earliest endocardial breakthrough during sinus rhythm (SR) occurred more frequently in the septal (63%, principally posteroseptal) than anterosuperior (37%) LA and varied little with isoproterenol or high right atrial pacing rate [21].

Findings of our study suggest that, multiple RF ablations at the low interatrial septum cause prolongation of IACT by affecting the posterior inter-atrial fibers. Our observation that right to left IACT prolonged after ablation but the left to right IACT remained constant supports the proposal of Markides et al. This can be explained by the newly understood importance of the posterior inter-atrial fibers in the activation of left atrium and the close proximity between low inter-atrial septum and Koch’s triangle.

## Clinical Implications

The present results may have clinical implications regarding atrial arrhythmogenesis. In this study; postablation inter-atrial conduction time increased significantly following RF ablation of the low inter-atrial septum. The role of this augmented conduction delay in the occurrence of late atrial arrhythmias needs to be evaluated in prospective studies.

## Study Limitations

The major technical difficulty encountered in the present study was the inability to achieve extensive mapping of the anterior and antero-septal aspects of the mitral annulus. For ethical reasons, the authors did not consider trans-septal or retrograde arterial approach in this study. Furthermore, left atrial activation was mostly represented by coronary sinus electrograms that provided information on the region of the left atrium adjacent to the mitral annulus. Given the complexity of septal activation pattern, simultaneous multisite mapping techniques (non-contact mapping of the CARTO system) are required for more accurate study of the interatrial electrical connections [22].

## Conclusion

RF ablation of AVNRT results in prolongation of IACT. The degree of prolongation is associated with the duration and number of RF ablations given. The relationship between this conduction delay and late arrhythmogenesis need to be evaluated.

## List of abbreviations used in the manuscript

RF: radiofrequency
AVNRT: atrioventricular nodal reentrant tachycardia
IACT: inter-atrial conduction time
SP: slow pathway
EPS: electrophysiologic study
